# Spectroscopic Investigations of the Binding Interaction of a New Indanedione Derivative with Human and Bovine Serum Albumins

**DOI:** 10.3390/molecules14041614

**Published:** 2009-04-24

**Authors:** Dana Stan, Iulia Matei, Carmen Mihailescu, Mihaela Savin, Mihaela Matache, Mihaela Hillebrand, Ion Baciu

**Affiliations:** 1 Department of Organic Chemistry, Bucharest University, 00133, Sos. Panduri No. 90-92, Romania; E-mails: carmen_mihail28@yahoo.com (C.M.), mihaela.matache@gmail.com (M.M.), ion_baciu2004@yahoo.com (I.B.); 2 Department of Physical Chemistry, Faculty of Chemistry, University of Bucharest, Bd. Regina Elisabeta, No. 4-12, Bucharest, Romania; E-mails: iulia.matei@yahoo.com (I.M.), mihaela.hillebrand@gmail.com (M.H.); 3 Telemedica, Str. Ion Calin, No.13, Bucharest, Romania; E-mail: mihaelasvn@yahoo.com (M.S.)

**Keywords:** Indanedione, Serum albumin, Fluorescence, Circular dichroism, Fluorescence resonance energy transfer

## Abstract

Binding of a newly synthesized indanedione derivative, 2-(2-hydroxy-3-ethoxybenzylidene)-1,3-indanedione (HEBID), to human and bovine serum albumins (HSA and BSA), under simulated physiological conditions was monitored by fluorescence spectroscopy. The binding parameters (binding constants and number of binding sites) and quenching constants were determined according to literature models. The quenching mechanism was assigned to a Förster non-radiative energy transfer due to the HEBID-SA complex formation. A slightly increased affinity of HEBID for HSA was found, while the number of binding sites is approximately one for both albumins. The molecular distance between donor (albumin) and acceptor (HEBID) and the energy transfer efficiency were estimated, in the view of Förster’s theory. The effect of HEBID on the protein conformation was investigated using circular dichroism and synchronous fluorescence spectroscopies. The results revealed partial unfolding in the albumins upon interaction, as well as changes in the local polarity around the tryptophan residues.

## 1. Introduction

Serum albumins (SAs) are the major soluble protein constituents of the circulatory system and have many physiological functions, including acting as transporters for numerous endogenous and exogenous ligands (e.g. drugs, fatty acids, etc.). Drug binding to SAs is an important determinant of drug pharmacokinetics, restricting the unbound concentration and affecting drug distribution and elimination [1]. 

Human serum albumin (HSA) is the major protein component of blood plasma (approx. 60% of the total protein) [2]. It is a single, non-glycosylated polypeptide that organizes to form a heart-shaped protein with approximately 67% α-helix [3]. HSA is capable of binding reversibly a wide variety of drugs, resulting in an increased solubility in plasma, decreased toxicity, and/or protection against oxidation of the bound ligand. The protein’s capability of binding aromatic and heterocyclic compounds largely depends on the existence of two major binding regions, namely Sudlow’s sites I and II [4]. Site I, also known as the warfarin binding site, is formed by a pocket in subdomain IIA and contains the only tryptophan of HSA (Trp 214) [5]. Site II is located in subdomain IIIA and is known as the benzodiazepine binding site [5]. Of these sites, site I seems to be the more versatile, because it can bind, with a high affinity, to ligands that are very different from a chemical point of view [2].

Bovine serum albumin (BSA) is constituted by 582 amino acid residues and, on the basis of the distribution of the disulfide bridges and of the amino acid sequence, it can be regarded as composed of three homologous domains (I, II and III) linked together. Each domain can be subdivided into two subdomains, A and B. BSA has two tryptophans, Trp-134 and Trp-212, embedded in subdomains IB and IIA, respectively [6].

During the last three decades, substituted indane-1,3-dione derivatives have shown wide applicability in the fields of medicine [7,8] and biology [9]. Many studies on the biological activity of these compounds report their anticoagulant [10], anti-inflammatory [11], analgesic [12], antibacterial [13] or bronchial dilating [14] action. However, literature data on their interaction with proteins is relatively scarce [15,16,17]. Because they are compounds of important biological activity, we considered that such studies could elucidate important aspects of drug pharmacokinetics.

In the present report we focus on a newly synthesized indanedione derivative, 2-(2-hydroxy-3-ethoxybenzylidene)-1,3-indanedione (HEBID), studying its interaction with human and bovine serum albumins (HSA and BSA) by means of fluorescence and circular dichroism spectroscopies. Fluorescence is a practical method for studying protein interactions with various ligands [18,19,20,21,22,23,24], as it yields a vast amount of information on the magnitude of binding and on the microenvironment surrounding the protein residues. In our work, we monitored the changes in the intrinsic fluorescence of the SAs upon binding to HEBID. A circular dichroism study was also undertaken in order to complement the findings by fluorescence spectroscopy, allowing us to ascertain the influence of HEBID binding upon the secondary structures of SAs. Therefore, the aim of this study was to characterize the HEBID-HSA and HEBID-BSA interactions, by determining the binding parameters, the efficiency of energy transfer and the conformational changes occurring in the proteins upon binding.

## 2. Results and Discussion

### 2.1. Fluorescence quenching of serum albumins in presence of HEBID

**Figure 1:**
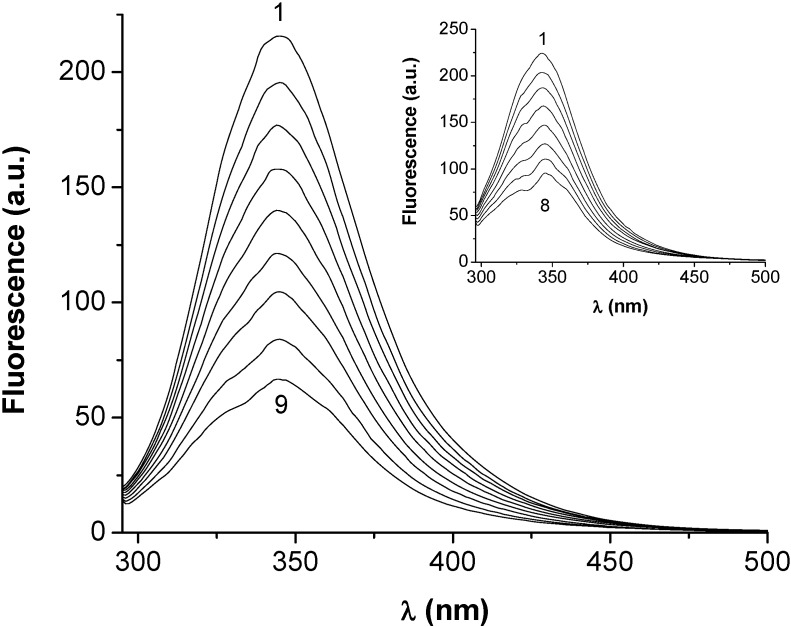
The quenching of BSA and HSA (inset) fluorescence at increasing HEBID concentrations. [HEBID]/[SA] = 0 (1), 0.48 (2), 0.97 (3), 1.67 (4), 2.53 (5), 3.83 (6), 5.19 (7), 7.61 (8), 10.00 (9). *λ_ex_* = 286 nm.

The fluorescence of HSA and BSA mainly resides in the emissions from the tryptophan, Trp (~340 nm) and tyrosine, Tyr (~315 nm) residues. Upon excitation at 286 nm, HSA and BSA show strong emissions, with peaks at 340 nm and 345 nm, respectively. It was observed that the fluorescence emission intensities of both SAs were quenched, in a concentration-dependent manner, when the proteins interacted with HEBID (a typical example is shown in Figure 1). 

**Figure 2:**
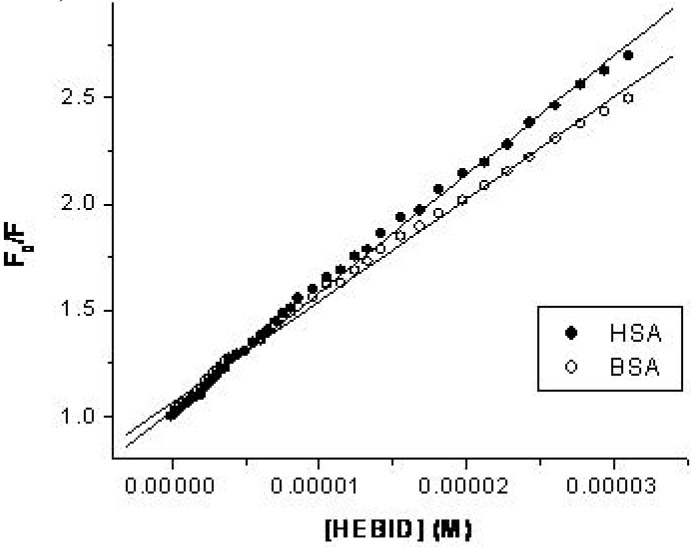
Stern-Volmer plots for the quenching of HSA and BSA by HEBID in the d/p range 0-10. *F_0_* and *F* denote the fluorescence intensities in the absence and presence of the quencher (*λ_ex_* = 286 nm).

Quenching of the intrinsic protein fluorescence can be used to retrieve many ligand-protein binding information. Fluorescence quenching is described by the well-known Stern-Volmer equation [25]:

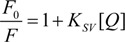
(1)
where *F_0_* and *F* denote the steady-state fluorescence intensities in the absence and presence of the quencher (HEBID), respectively, *K_SV_* is the Stern-Volmer quenching constant and [*Q*] the quencher concentration. Hence, Equation (1) can be applied to determine *K_SV_* by linear regression of a plot of *F_0_*/*F* against [*Q*] (Figure 2). *K_SV_* was estimated at 5.55×10^4^ M^-1^ for the HEBID-HSA interaction (correlation coefficient R = 0.9988) and at 4.80×10^4^ M^-1^ for the HEBID-BSA interaction (R = 0.9976).

### 2.2. Estimation of the binding parameters

**Figure 3:**
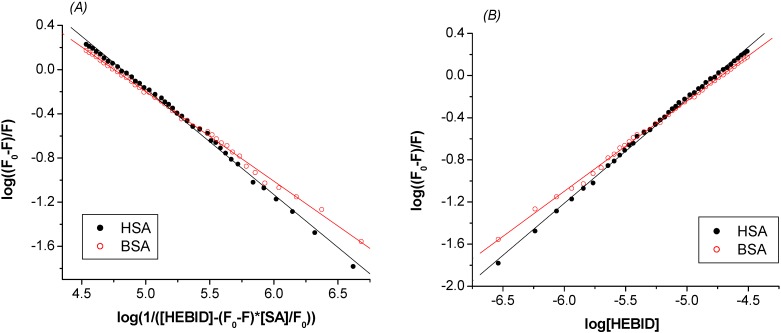
Plots for the determination of the binding parameters, according to: (A) Equation (2) and (B) Equation (3). [SA] = 3×10-6 M, λex = 286 nm, λem = 335nm.

The estimation of the binding parameters was made using two formulae that consider the presence of a single class of binding sites. The first formula, Equation (2), starts from the classical Scatchard equation [26] and expresses the free ligand concentration at equilibrium as a function of the analytical, total concentration of the ligand [27,28]:

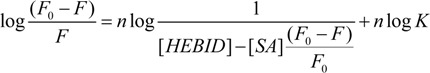
(2)
*K* and *n* are the binding constant and number of binding sites, and [HEBID] and [SA] are the total ligand concentration and the total protein concentration, respectively. Linear fits according to Equation (2), i.e. log(*F_0_*-*F*)/*F* vs. log(1/([HEBID]-[SA](*F_0_*-*F*)/*F_0_*)), are presented in Figure 3. The binding parameters, evaluated from the slope and intercept of these plots, are listed in Table 1. It can be seen that *n* is about 1 for both SAs, suggesting that one molecule of albumin combines with one molecule of HEBID in the drug to protein (d/p) molar ratio under study, ranging from 0 to 10. We can also observe that the binding affinity of HEBID to HSA is slightly higher than that to BSA.

The second formula, equation (3), is based on the approximation that the free ligand concentration, at equilibrium, can be replaced by the total analytical concentration of the ligand, approximation that is only valid when the ligand is in excess, like in our working conditions [29,30].

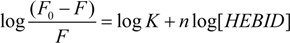
(3)


It can be observed from Table 1 that, in our case, both equations lead to similar values of the binding constants and attest a one to one ligand-protein interaction, implying the binding of the ligand to the Trp 214 residue.

**Table 1:** Binding parameters (binding constants, K, and number of binding sites, n) characterizing the HEBID-HSA and HEBID-BSA interactions, estimated in the d/p range 0-10.

		*K* (M^-1^) ×10^-4^	*n*	R	SD
**Equation (2)**	**HSA**	6.54	0.96	0.9985	0.0279
	**BSA**	5.65	0.81	0.9989	0.0209
**Equation (3)**	**HSA**	5.47	0.99	0.9987	0.0262
	**BSA**	1.11	0.86	0.9992	0.0180

### 2.3. Effect of HEBID on the conformations of HSA and BSA

#### 2.3.1 Synchronous fluorescence measurements

**Figure 4:**
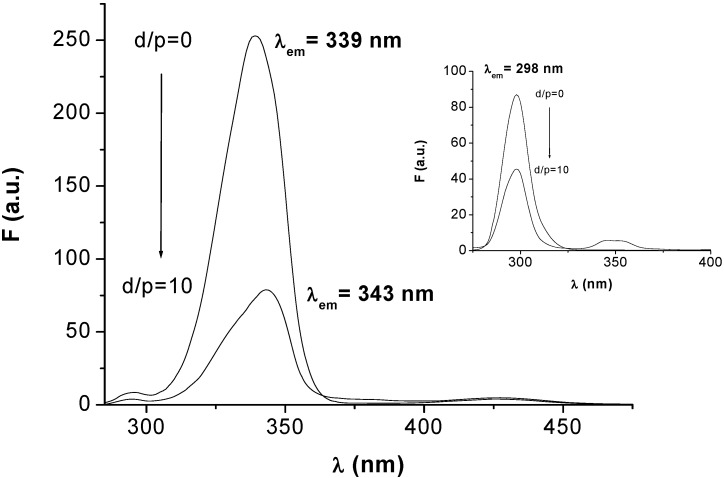
The HEBID effect on the synchronous fluorescence emission of HSA (Δλ = 60 nm). Inset: Δλ = 15 nm.

Synchronous fluorescence is a method that provides information on the molecular environment in the vicinity of the fluorophores of SAs. While the sensitivity of the fluorescence method is preserved, this technique offers several advantages, such as spectral simplification, bandwidth reduction and therefore a minimization of the effects that interfere in the steady-state technique [31,32]. Using *Δλ* = 60 nm and 15 nm we obtain the characteristic synchronous fluorescence spectra of the Trp and Tyr residues, respectively. The shifts in the position of the synchronous maxima of these residues, which usually occur upon binding, give indications on the changes in polarity around these particular fluorophores and thus on the proximity of the ligand. Figure 4 shows the effect of HEBID addition on the position of the synchronous emission maxima of the Trp and Tyr residues of HSA.

As evidenced in Figure 4, the emission maximum of Tyr does not show a significant shift upon binding, which indicates that the polarity around the Tyr residues of HSA remains unchanged. Differently, the red shift of approximate 4 nm in the emission maximum of the Trp residue suggests that the HEBID-HSA interaction results in a more polar environment for the Trp residue. A similar trend was observed for BSA.

#### 2.3.2. Circular dichroism results

When a ligand binds to a globular protein, the intramolecular forces responsible for maintaining the secondary and tertiary structures can be altered, resulting in a conformational change of the protein [33]. The observed fluorescence quenching of the Trp and Tyr residues suggests that the ligand-albumin interaction has indeed changed the microenvironment of these residues. Moreover, the red shift in the synchronous fluorescence of Trp shows an increased polarity of the microenvironment after binding.

**Figure 5:**
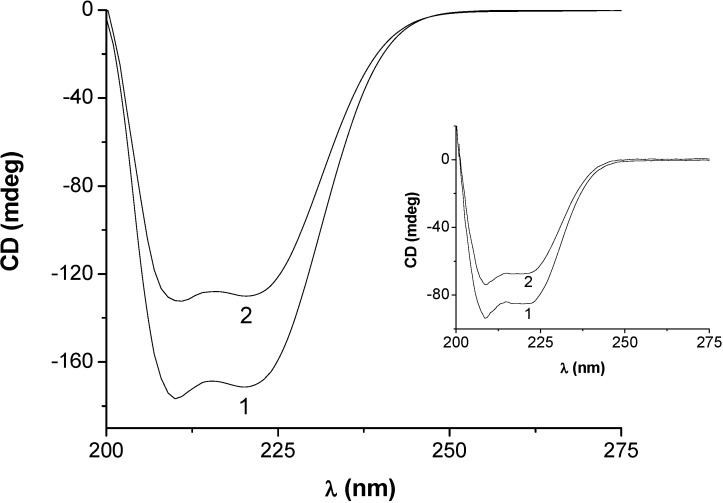
The CD spectra of HEBID-HSA and HEBID-BSA (inset) systems. [HSA] and [BSA] = 0.75×10-6 M. [HEBID] = 0 M (1) and 1.38×10-5 M (2). pH = 7.4.

Conformational changes upon binding are also reflected in the circular dichroism (CD) spectrum of the protein. The CD spectra of SAs exhibit two negative bands in the UV region at 209 and 222 nm, characteristic for the α-helical structure [34]. The spectra recorded in presence of low HEBID concentrations did not exhibit appreciable changes in the conformation of SAs in terms of α-helicity. However, significant changes in α-helicity values were noticed at higher concentrations of HEBID. The CD spectra of HSA and BSA in the absence (d/p = 0) and presence (d/p = 18) of HEBID are shown in Figure 5. The alterations of protein secondary structure after HEBID complexation were estimated as follows. The results of CD measurements can be expressed as MRE (mean residue ellipticity) in deg cm^2^ dmol^-1^, defined as:

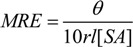
(4)
where *θ* is the observed CD (in milli-degrees), *r* is the number of amino acid residues (585 for HSA and 582 for BSA), and *l* is the path length of the cell (in cm). The helical content of free and bound SA was calculated from the MRE values at 209 nm using the following equation, as described by Lu et al. [35]:

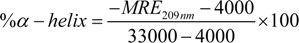
(5)


It was observed that the percent of α-helix was reduced from 59.98% in free BSA to 57.58 % upon binding to HEBID (d/p =18). The fact that the CD spectra of BSA in the presence and absence of HEBID are similar in shape indicates that the structure of BSA remains predominantly α-helical after binding to HEBID. Similarly, for HSA, the protein α-helix structure decreased gradually and the reduction reached about 5% for protein binding with HEBID at a d/p = 18, indicating partial unfolding of HSA.

### 2.4. Energy transfer efficiency and binding distance

**Figure 6:**
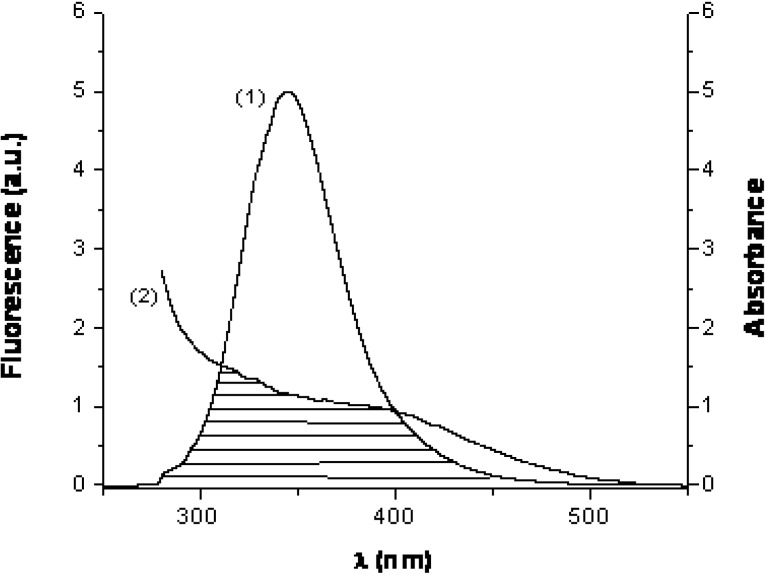
Overlap of the fluorescence spectrum of BSA (1) and absorption spectrum of HEBID (2).

The performed spectral studies suggested that both HSA and BSA form complexes with HEBID, with involvement of the Trp residues of the proteins. HSA possesses only one Trp molecule (Trp214), while BSA contains two such residues (Trp134 and Trp212), though only Trp134 is readily accessible to the ligand, being located on the exterior surface of the protein [36]. A way of measuring the distance *r* between the ligand and these Trp residues is by using the fluorescence resonance energy transfer (FRET), as proposed by Förster [37]. FRET is a distance dependent interaction in which excitation energy is transferred nonradiatively from the donor molecule to the acceptor. The prerequisite for the occurrence of FRET is the existence of a proper overlap between the emission spectrum of the donor and the absorption spectrum of the acceptor. Figure 6 shows this overlap in the case of the HSA-HEBID donor-acceptor pair.

The extent of the spectral overlap, as well as the distance between donor and acceptor, determine the magnitude of the energy transfer. According to Förster, the energy transfer efficiency, *E*, is given by:

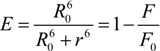
(6)
where *r* is the distance between donor and acceptor and *R_0_* is the critical distance, for which the transfer efficiency is 50%. *F_0_* and *F* are the fluorescence intensities of HSA in absence and presence (at d/p = 1) of HEBID, respectively. The magnitude of *R_0_* depends on the spectral properties of the donor and acceptor molecules:

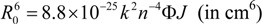
(7)


Here *k* is a factor expressing the spatial orientation of the dipole of the acceptor molecule, *n* is the refractive index of the medium, Φ the fluorescence quantum yield of the donor in absence of the acceptor and *J* is the overlap integral of the emission spectrum of the donor and absorption spectrum of the acceptor (in units of M^-1^cm^3^). *J* is given by the equation:

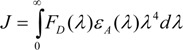
(8)
where *F_D_(λ)* is the corrected fluorescence intensity of the donor at wavelengths *λ* to (*λ* + *Δλ*), with the total intensity normalized to unity, and *ε_A_(λ)* is the molar extinction coefficient of the acceptor at wavelength *λ*.

The efficiency of the dipole-dipole interaction between donor and acceptor depends on the alignment of the two dipoles. The orientation factor *k^2^* which describes this proximity ranges from 0 (perpendicular dipoles) to 4 (parallel dipoles). Generally, the dipoles are assumed to be rapidly moving, on timescales similar to the donor excited-state lifetime, and their orientations are therefore described as random, with an orientation factor of 2/3 [38].

Förster’s distance (*R_0_*) has been calculated assuming such random orientation of the donor and acceptor molecules in solution. Thus, *k^2^* was taken 2/3, *n* = 1.333 (water) and *Φ* = 0.118 [39]. The obtained data is listed in Table 2.

**Table 2:** Förster energy transfer parameters.

	*E*	*J* × 10^15^ (M^-1^cm^3^)	*R_0_* (nm)	*r* (nm)
**HSA**	0.16	1.48	1.78	2.34
**BSA**	0.18	1.51	1.79	2.31

An average *r* distance on the 2-8 nm scale indicates that the energy transfer occurs with high probability [40]. Furthermore, a larger *r* value than that of the critical distance *R_0_* suggests the presence of a static quenching mechanism [41].

## 3. Experimental

### 3.1. General

The IR spectra were recorded on a Fourier Transform Genesis DTGS in KBr pellets in the range 4000-400 cm^-1^. The NMR spectra were recorded on a JEOL Lambda 400 (operating at 300 MHz for ^1^H and 75 MHz for ^13^C, respectively) in DMSO-d_6_, using TMS as internal standard. Mass spectra were obtained on a Finnigan MAT 90 spectrometer using CI technique; Thin Layer Chromatography was carried out on silicagel plates (Merck) and visualisation was accomplished using iodine vapours or UV light (*λ* = 254 nm). The melting points were determined in open capillaries using an electrical melting point apparatus and are uncorrected. The absorption and fluorescence spectra were recorded on a V-560 Jasco UV-VIS spectrophotometer and on a FP-6300 Jasco spectrofluorimeter equipped with a STR-313 Jasco thermostatic cell holder, respectively. Circular dichroism measurements were carried out with a Jasco J-815 CD spectrometer. All chemicals were obtained from commercial sources and used without further purification. Human and bovine serum albumins were fatty acids free, 99%.

### 3.2. Synthesis

**Scheme 1:**
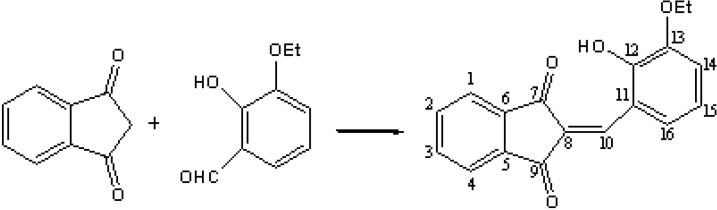
Synthesis of the arylidene derivative.

The synthesis of 2-(2-hydroxy-3-ethoxybenzylidene)-1,3-indanedione (HEBID) is based on the direct condensation between 1,3-indanedione and aromatic aldehydes, as depicted in Scheme 1. 2-Hydroxy-4-ethoxybenzaldehyde (3.32 g, 20 mmoles) and glacial acetic acid (50 mL) were added to 1,3-indanedione (2.9 g, 20 mmoles) [42]. The mixture was heated and when it became clear, concentrated hydrochloric acid (1 mL) was added. The mixture was heated for 20 minutes at reflux, then allowed to cool to room temperature. The solid formed was filtered and washed with ethanol (15 mL) to affird 4.7 g of an orange product (yield 80%); R_f_ = 0.37 (chloroform-methanol-petroleum ether 4:1:2 v:v:v). It was recrystallized from ethanol to yield crystals with m.p. 221-222 ºC. ^1^H-NMR (δ ppm): 9.84 (s, 1H, OH); 8.47 (d, 1H, J=8, H14); 8.36 (s, 1H, H10); 7.91-7.98 (m, 4H, H1-H4); 7.20 (d, 1H, J=8, H16); 6,68 (t, 1H, J=8, H15); 4.12 (q, 2H, J=7, CH2O); 1.38 (t, 3H, J=7, Me). ^13^C-NMR (δ ppm): 189.6, 188.4 (C7, C9); 149.5 (C13); 146.4 (C12); 141.5 (C10), 139.4, 139.0 (C5, C6); 135.5, 135.3 (C2, C3); 127.0, 124.0 (C11, C8); 122.6, 122.4 (C1, C4); 119.9 (C15) 118.2 (C16), 117.8 (C14); 64.2 (CH2O); 14.2 (Me); IR (ν cm^-1^): 3073, 1719, 1676, 1569, 1465, 1231; MS (m/z, %): calc. 294.3 ([M]^+^); found 294.9 (100, [M+H]^+^). 

### 3.3. Procedures

Protein solutions in phosphate buffer (pH 7.4) 3×10^-6^ M (for fluorescence studies) and 0.75×10^-6^ M (for CD studies) were prepared and equilibrated over night. A stock solution of HEBID in ethanol (1.2×10^-4^ M) was diluted with phosphate buffer to yield a 1.2×10^-5^ M solution used in the titration experiments. It should be noted that such low amounts of ethanol do not affect the protein structures [43].

Binding of HEBID to HSA and BSA was studied by the fluorescence quenching titration method, using the intrinsic fluorescence of the protein as a probe. To a fixed volume (2 mL) of protein solution were added increasing volumes of HEBID to obtain different complexes in a range of drug to protein (d/p) ratio of 0 to 10. Steady-state fluorescence spectra were obtained at excitation wavelength of 286 nm (excitation of Trp and Tyr residues). Synchronous fluorescence spectra were obtained using *Δλ* = 15 nm (Tyr) and 60 nm (Trp), where *Δλ* = *λ_em_* - *λ_ex_*. Circular dichroism spectra of SA and HEBID-SA complexes (d/p = 18) were scanned in the range 200-280 nm, in order to record the characteristic signal of the α-helix conformation of the albumins. All experiments were conducted at 25±1 ºC.

## 4. Conclusions

The binding interactions of HEBID with HSA and BSA were studied under simulated physiological conditions using steady state and synchronous fluorescence and circular dichroism spectroscopies. The results showed a significant interaction of HEBID with both HSA (*K* = 6.54×10^4^ M^-1^) and BSA (*K* = 5.65×10^4^ M^-1^), via one binding site for each protein. The effect of HEBID on the conformations of the SAs was also discussed, evidencing partial defolding of the proteins (2-5% loss in the α-helix content), caused by a perturbation of the Trp rather than of the Tyr residues. Binding distances of approximate 2 nm were computed, suggesting a high probability for the SA-HEBID energy transfer.
